# Posterior reversible encephalopathy syndrome in dengue virus infection with concurrent encephalitis and ischemic stroke: a case report

**DOI:** 10.1186/s12879-026-12920-8

**Published:** 2026-02-21

**Authors:** Dongdong Ren, Win Khaing, Mervyn Qi Wei Poh, Suma Sathyanarayana Rao, Yee-Sin Leo

**Affiliations:** 1 Department of Infectious Diseases, Tan Tock Seng Hospital, Singapore, 308433 Singapore; 2 Department of Infectious Diseases, Singapore General Hospital, Singapore, 169608 Singapore; 3 National Centre for Infectious Diseases, Singapore, 308442 Singapore; 4 Department of Neurology, National Neuroscience Institute, Singapore, 308433 Singapore; 5 Saw Swee Hock School of Public Health, National University of Singapore, Singapore, 117549 Singapore

**Keywords:** Case report, Dengue virus infection, Dengue encephalitis, Posterior reversible encephalopathy syndrome, Ischemic stroke, Neurological complications

## Abstract

**Background:**

Dengue is hyperendemic in Singapore and typically presents as an acute febrile illness. Neurological complications, such as dengue encephalitis and encephalopathy, are increasingly recognized; however, dengue-associated posterior reversible encephalopathy syndrome (PRES) remains rare.

**Case presentation:**

We report a 90-year-old woman with primary dengue virus serotype 3 infection who developed persistent altered mental status during the critical phase of illness. Neuroimaging revealed bilateral asymmetric parieto-occipital vasogenic edema with focal hemorrhage, consistent with PRES, together with small acute infarcts in the deep watershed and left middle cerebral artery territories. Cerebrospinal fluid analysis demonstrated mildly elevated protein without pleocytosis; dengue IgM and IgG were positive, while dengue PCR was negative. She was managed with supportive care with close neurological monitoring. Follow-up imaging at two months showed near-complete radiological resolution accompanied by significant clinical improvement.

**Conclusion:**

This case highlights two important considerations. First, altered mental status in elderly patients with dengue should not be attributed solely to delirium, and prompt neurological evaluation is essential. Second, dengue infection may be associated with overlapping neurological manifestations, including dengue encephalitis, PRES and ischemic stroke. Recognition of such overlap is important for accurate diagnosis and management of dengue-related central nervous system involvement.

**Clinical trial number:**

Not applicable.

## Background

Dengue is hyperendemic in Singapore, with more than 13,000 cases reported in 2024. Dengue virus (DENV) infection typically presents with acute onset of fever, rashes, myalgia, arthralgia and lethargy, and severe disease may be complicated by hemorrhagic shock. Historically, dengue virus was considered to be non-neurotropic virus [1], however, a recent review estimated that neurological manifestations occur in 0.5–21% of hospitalized dengue cases [2], including dengue encephalopathy, dengue encephalitis, immune-mediated syndromes, neuromuscular dysfunctions and neuro-ophthalmic disorders [2], particularly in DENV-2 and DENV-3 infections [1].

The neuropathogenesis of dengue remains poorly understood, and likely mechanisms include direct central nervous system (CNS) invasion, immune-mediated injury and metabolic alterations [1, 3]. While dengue encephalitis is increasingly recognized, posterior reversible encephalopathy syndrome (PRES) has been rarely reported in association with dengue infection [1]. Although both conditions may present with altered mental status (AMS) or focal neurological deficits, they are considered distinct entities with different pathophysiology, cerebrospinal fluid (CSF) and neuroimaging characteristics. Nevertheless, in the setting of severe dengue with capillary leakage, overlapping clinically and radiological features may occur.

Here, we report a 90-year-old woman with DENV-3 infection who developed dengue-associated PRES, encephalitis and concurrent ischemic stroke. To our knowledge, this is the oldest reported case of dengue-associated PRES. This case highlights that dengue-related neurological complications may overlap and occur even in the absence of typical risk factors such as severe hypertension, autoimmune disease or immunosuppressive therapy. It also emphasizes the importance of considering dengue-related central nervous system involvement in elderly patients, in whom AMS is often misattributed to delirium.

## Case presentation

A 90-year-old Chinese woman with a history of hypertension, bilateral knee osteoarthritis, and erosive gastritis was admitted for five days of fever, myalgia and reduced oral intake. She had previously been independent in daily activities, though family members reported mild amnestic cognitive impairment over the preceding months.

On admission (day 5 of illness), she was febrile and lethargic. Blood pressure was 163/71mmHg at supine and 145/65mmHg while seated. Physical examination was unremarkable and there were no dengue warning signs. Initial laboratory investigations showed a white blood cell count of 3.6 × 10^9^/L, hemoglobin of 11.9 g/dL, hematocrit of 36.6%, platelets count of 60 × 10^9^/L, and mildly elevated aspartate aminotransferase of 91 U/L. Dengue NS1 antigen (an antigenic marker of early dengue infection), performed on day 4 of illness at a private clinic was positive, while IgM and IgG were negative, consistent with primary dengue virus infection. Her serum creatinine was elevated at 131 µmol/L, and low-volume intravenous fluids were administered for acute kidney injury.

On day 6 of illness, her hematocrit rose to 42.4% (a 15.8% increase), and platelet count reached a nadir of 21 × 10^9^/L. That night, she developed AMS characterized by disorientation, agitation, and behaviours disinhibition including pulling out intravenous plug and playing with stool.

By day 7 of illness, she remained persistently confused and uncooperative despite improving platelet count (25 × 10^9^/L). A delirium workup -including vitamin B12, folate, thyroid function, renal function and electrolytes, liver function, HIV and syphilis serology- was unremarkable.

Blood pressure was monitored closely during the acute illness, and fluctuated within the range of 120-160mmHg systolic, without documented hypotension or severe hypertension.

**Fig. 1:**
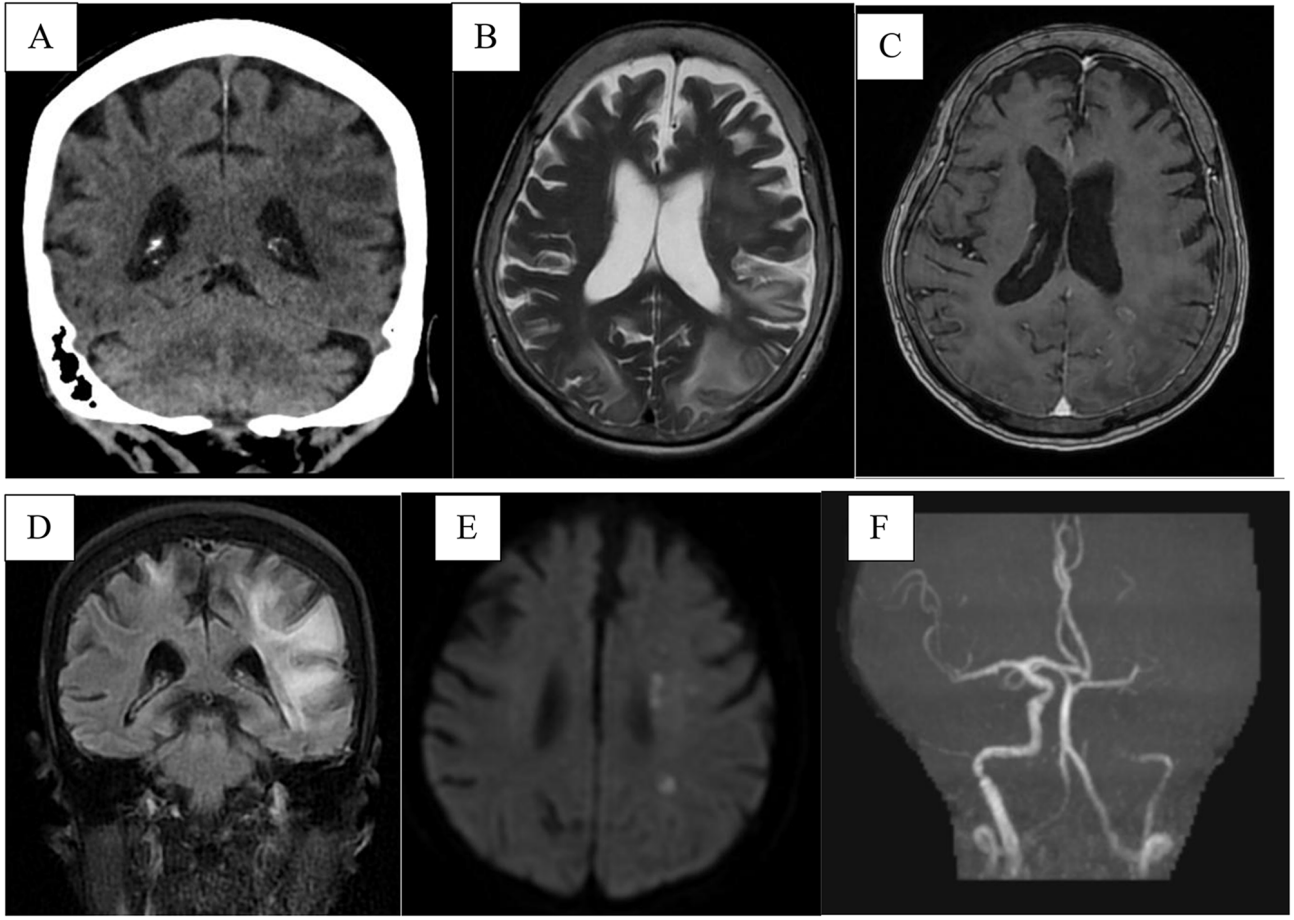
Neuroimaging findings during acute dengue illness. (**A**) Non- Contrasted CT brain showing bilateral parieto-occipital hypodensities. (**B**) MRI T2W image demonstrating confluent areas of hyperintensities over deep and subcortical white matter bilaterally in the parieto-occipital lobes, more pronounced on the left, extending to the left temporal and left frontal lobes. (**C**) Post-contrast T1-weighted image showing mild leptomeningeal enhancement in the left parieto-occipital lobes and small foci of parenchymal enhancement in the left parietal deep white matter. (**D**) FLAIR demonstrating hyperintensities at the cortical/subcortical regions of bilateral parieto-occipital lobes, asymmetrically more on the left. (**E**) Diffusion-weighted image showing multiple scattered small foci of restricted diffusion over left deep white matter, consistent with acute infarcts in the deep watershed territory and left MCA territory. (**F**) MR angiography demonstrating severe left internal carotid artery and left middle cerebral artery stenosis

Non-contrast computed tomography (CT) of the brain on day 8 of illness showed bilateral parieto-occipital vasogenic edema (Figue 1 A). Subsequent magnetic resonance imaging (MRI) revealed confluent bilateral parieto-occipital T2-weighted (T2W)/fluid-attenuated inversion recovery (FLAIR) hyperintensities, more prominent on the left, with associated petechial hemorrhages extending into the left temporal and frontal lobes. Mild leptomeningeal enhancement and additional parenchymal signal changes were also observed (Fig. 1B-D). Diffusion-weighted imaging demonstrated small acute infarcts in the left deep watershed and middle cerebral artery territories with magnetic resonance angiography showing severe steno-occlusion of the left internal carotid artery with poor/little flow in the left middle cerebral artery (Fig. 1E-F).

Electroencephalogram (EEG) showed continuous diffuse slow activity with absence of normal background rhythm, consistent with severe diffuse encephalopathy. CSF analysis revealed 19 red blood cells/µL, < 1 nucleated cell/µL and mildly elevated protein at 0.48 g/L. CSF Dengue IgM and IgG were positive, while dengue PCR was negative. Serum dengue PCR confirmed dengue virus serotype 3. Additional CSF studies including tetraplex PCR, FilmArray meningitis/encephalitis panel, cytology, and flow cytometry were negative.

She was transferred to the neurology ward for close neurological monitoring and supportive care. Aspirin was initiated following platelet recovery, taking into account the presence of petechial hemorrhage. Blood pressure was managed conservatively to avoid relative hypotension in view of watershed infarct with left internal carotid and middle cerebral artery stenosis. A nasogastric tube was inserted because of poor oral intake. After approximately one month of hospitalization, she was discharged to a nursing facility.

**Fig. 2:**
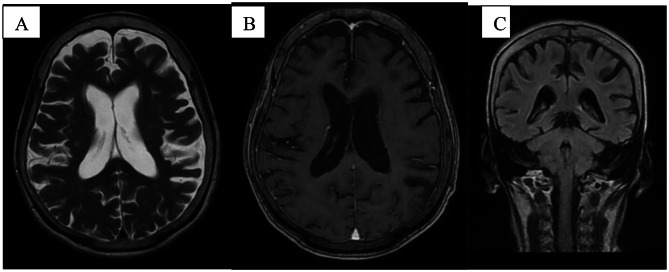
Neuroimaging findings during follow-up in 2months. (**A**) MRI T2W image showing interim resolution of prior parieto-occipital hyperintensity changes. (**B**) Post-contrast T1-weighted demonstrating resolution of leptomeningeal enhancement in the left parieto-occipital lobe. (**C**) FLAIR sequence showing resolution of previous hyperintensities over bilateral parieto-occipital lobes

At two-month follow-up, repeat MRI demonstrated near-complete resolution of prior white matter changes and leptomeningeal enhancement (Fig. 2A-C). Clinically, she showed significant improvement in alertness and behaviour. The nasogastric tube had been removed as she regained the ability to swallow and resume oral intake. She was eating and sleeping well, recognized immediate family members, and was able to engage in simple conversation. Agitation had resolved, and no further ongoing behaviour disturbances were observed. At longer term follow-up, she maintained ambulant and functionally independent but had gradual declining of cognitive impairment. A diagnosis of Alzheimer’s disease was made based on longitudinal cognitive history and she was started on donepezil. A detailed chronological summary of clinical events, laboratory trends, neuroimaging findings and follow-up is provided in Table 1.

**Table 1:**
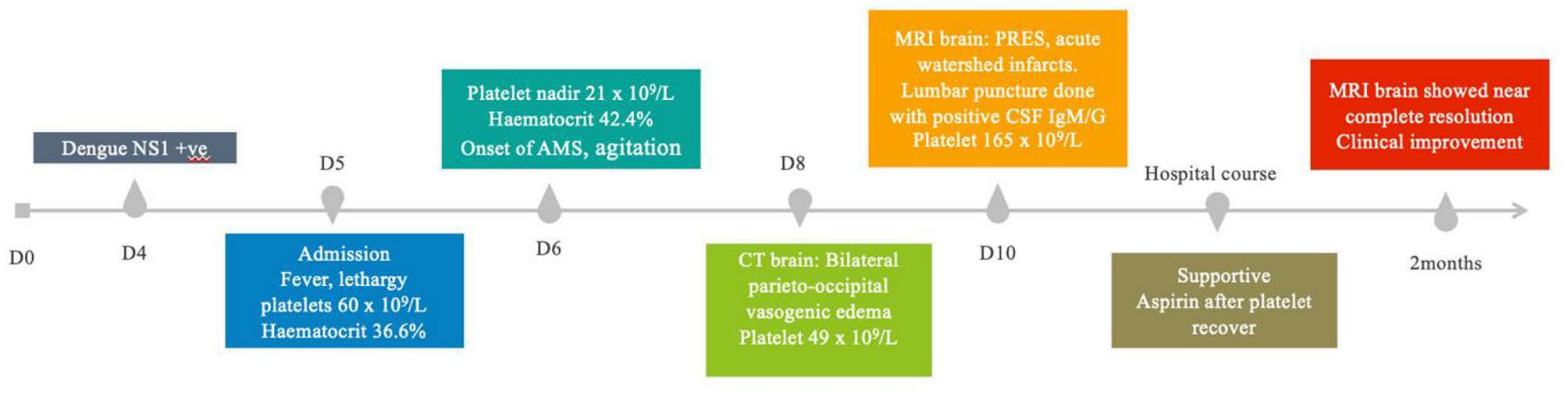
Timeline of clinical course and neuroimaging findings

## Discussion and conclusions

Neurological complications following DENV infection are increasingly recognized and encompass a heterogenous spectrum of clinical and radiological manifestations, mediated by either direct virus neuro-invasion or immune-related injury or systemic metabolic disturbance. Dengue encephalopathy and encephalitis are the most commonly described entities [1, 2], while posterior reversible encephalopathy syndrome has emerged as a rare but increasingly reported neurological presentation in recent years. Importantly, these syndromes are not mutually exclusive, and may have overlapping clinical, radiological, and CSF features, which complicate the diagnosis and management.

Our case described an elderly patient with DENV-3 infection presenting with multiple neurological syndromes-dengue encephalitis, PRES, and concomitant ischemic stroke-illustrating the challenges of attributing neurological manifestation to a single pathological process.

### Differentiating dengue encephalopathy, dengue encephalitis, PRES and ischemic stroke

Dengue encephalopathy is generally attributed to systemic or metabolic disturbances- such as hepatic failure, renal failure, electrolytes imbalance, or shock, and is typically associated with normal CSF and neuroimaging [2].

In contrast, dengue encephalitis is believed to result from direct viral invasion of the brain with viral replication and cytokine-mediated inflammation [4]. Clinically, it commonly manifests with altered mental status, seizures or focal neurological deficits [5]. The diagnosis is confirmed by detection of DENV in the CSF, including NS1 antigen, dengue PCR or anti-DENV IgM [6]. However, CSF pleocytosis is inconsistently present, and normal CSF cellularity has been shown in more than half of cases [7].

Radiologically, dengue encephalitis most commonly presents as diffuse cerebral edema or bilateral symmetrical FLAIR/T2W hyperintensities involving the thalamus, basal ganglia, pons and cerebellum. Diffusion restriction reflecting cytotoxic edema and meningeal enhancement may be present. Petechial hemorrhage has also been described [6, 8]. The “double doughnut sign” is a recognized but uncommon finding [9]. EEG typically demonstrates diffuse slowing [6].

Posterior reversible encephalopathy syndrome is a clinical-radiological entity characterized by acute or subacute neurological symptoms- including encephalopathy, confusion, headache, visual disturbances and seizure- associated with bilateral symmetrical, predominantly parieto-occipital vasogenic edema on neuroimaging [10–12]. It is classically associated with severe hypertension, renal failure, eclampsia, autoimmune disease, or exposure to cytotoxic or immunosuppressive agents. Infection triggers, including COVID and Lyme neuroborreliosis, have also been reported [10]. Beyond blood pressure dysregulation, endothelial dysfunction triggered by toxins or cytokines is increasingly recognized as a key contributor to PRES pathophysiology [10]. With appropriate management, clinical and radiological recovery are usually favourable.

In our patient, differentiation between these entities was challenging. Altered mental status developed during the dengue critical phase, coinciding with nadir thrombocytopenia and hemoconcentration, and was initially attributed to delirium related to acute illness. Subsequent neuroimaging and CSF evaluation revealed findings that could not be explained by a single diagnosis.

The detection of anti-DENV IgM in the CSF, fulfilled the diagnostic criteria of confirmed encephalitis according to International Encephalitis Consoritum [13]. Although the absence of CSF pleocytosis and negative CSF dengue PCR were atypical, these findings may reflect late-phase disease with reduced viral burden in the CSF or assay limitation. Normal CSF cellularity has been well described in dengue encephalitis.

Concurrently, MRI demonstrated bilateral parieto-occipital vasogenic edema. This imaging pattern was less typical of classical dengue encephalitis, which more commonly presents with cytotoxic edema and thalamic or basal ganglia involvement. Instead, the posterior circulation -predominant vasogenic edema and subsequent radiological and clinical reversibility were more in keeping with PRES. Notably, she lacked classical triggers such as severe hypertension, autoimmune disease, or exposure to immunosuppressant. Her blood pressure fluctuated between 120-160mmHg without documented hypotension or severe hypertension, suggesting that mechanism beyond blood pressure extremes may have contributed to PRES development.

The small acute infarcts in the deep watershed and left middle cerebral artery territories were most likely related to underlying severe chronic left internal carotid artery/middle cerebral artery stenosis. During the dengue critical phase, plasma leakage and hemoconcentration may have predisposed the already compromised vascular territory vulnerable to relative hypoperfusion. However, ischemic stroke alone would not be able to explain the vasogenic edema or CSF anti-DENV IgM positivity. These observations support the co-existence of multiple overlapping pathological processes rather than a single diagnosis.

### Management considerations

Management was similarly complex. Aspirin was only initiated after platelet recovery during the dengue recovery phase, balancing the presence of small petechial hemorrhages against the risk of recurrent ischemia stroke in the setting of severe internal carotid artery/middle cerebral artery stenosis. Single antiplatelet was chosen over dual antiplatelet therapy to minimize the bleeding risk.

Corticosteroids were not administered due to the absence of features suggestive of immune-mediated process (such as acute disseminated encephalomyelitis) and the lack of evidence supporting steroid use in dengue-associated PRES or encephalitis. Empirical acyclovir was given briefly while awaiting CSF viral studies and discontinued once results were negative. Given the presence of watershed infarction, permissive hypertension (systolic blood pressure < 180 mmHg) was allowed during the acute phase, and antihypertensive agent was withheld.

### Dengue-associated PRES: review of reported cases

Dengue-associated PRES was first reported in a 27-year-old male with headache and blurred vision by Sohoni et al. [14]. Since then, 11 more cases have been documented, primarily from dengue-endemic regions [15–25]. Our review of 12 reported cases (Table 2) demonstrates several consistent features. Most patients were young and female (83.3%), aged 8 to 68 years old, with 3 cases occurring during the late pregnancy. Classical PRES risk factors were largely absent. Seizures were the most common neurological manifestation, followed by altered mental status, headache, and visual disturbances. Blood pressure profiles were heterogenous, with hypotension and normotension observed as frequently as hypertension. CSF analysis, performed in approximately half of the cases, revealed mostly pleocytosis or elevated protein levels, with dengue IgM detected in 2 cases suggesting that overlap with dengue encephalitis may occur. Neuroimaging, predominantly MRI, typically demonstrated bilateral T2W/FLAIR hyperintensities involving the parieto-occipital regions with cortical and subcortical involvement. Compared with classical PRES cohorts, dengue-associated cases appear more likely to occur in patients without pre-existing cardiovascular risk factors and in the setting of severe dengue, suggesting a potentially distinct precipitating context. Outcomes were general favourable with supportive treatment.

**Table 2:** Overview of reported dengue-associated PRES cases

Author/Year	Age/sex	Comorbidities	Dengue diagnosis	Features of severe dengue	Blood pressure	PRES symptoms	CSF findings	Brain imaging	PRES management	Outcome
Sohoni et al. (2015) [14]	27/M	Nil	Dengue NS1, IgM positive	Nil	Normotensive	Headache, blurring of vision	Lymphocytic pleocytosis (85% lymphocytes), glucose 0.73 g/L, protein 1.02 g/L, Dengue IgM positive	MRI: Symmetrical gyral hyperintensities in bilateral parieto-occipital regions on T2W/FLAIR	Supportive treatment	Recovered
Nguyen et al.(2018) [15]	55/F	Nil	Dengue NS1 positive	Nil	Normotensive	Seizure AMS Slurred speech	Cell count of 7 /ul, protein 44 g/dl, Dengue IgM +ve, Dengue PCR -ve	MRI: Bilateral symmetrical hyperintensities on T2W/FLAIR over periventricular and deep cerebral white matter	Steroids, AEDs	Recovered
Sawant et al. (2020) [16]	10/F	Nil	Dengue NS1 positive	Yes, respiratory distress and hypotension, DIC	Hypertensive	Seizure	No red or white blood cell, protein 0.228 g/L, Dengue PCR -ve	MRI: Symmetric cortical and subcortical hyperintensities over bilateral frontal, parietal, temporal and occipital parenchyma	Antihypertensives; AEDs	Recovered
Biswas et al. (2024) [17]	20/F	37weeks pregnant	Dengue NS1 positive, IgM positive	Yes, respiratory distress, hypotension, DIC	Hypotensive	AMS, vision loss, seizure	Nil	MRI: Cortico-subcortical T2W/FLAIR hyperintense areas in posterior parietal and occipital regions, cerebellum.	AEDs Supportive treatment	Death; Intrauterine death
Sarkar et al. (2018) [18]	68/F	Nil	Dengue NS1 positive, IgM positive	Yes, hypotension	Hypotensive	AMS, seizure	Cell count of 15/ul (lymphocytic), protein 1.79 g/L	MRI: T2W/FLAIR hyperintensities in bilateral parieto-occipital cortex with subcortical white matter	AEDs	Recovered
Marakwad et al. (2022) [19]	8/F	Nil	Dengue NS1 positive	Nil	Hypertensive	Headache, seizure	Nil	MRI: Asymmetrical non enhancing areas of T2W/FLAIR hyperintensities in left parietal region	AEDs Anti-hypertensives	Recovered
Manya et al. (2021) [20]	15/F	Nil	Dengue NS1 positive	Yes, hypotension	Hypotensive	Seizure	Nil	MRI: Multiple non-enhancing near symmetrical patchy areas of T2W/FLAIR hyperintensities over cerebral and cerebellum	AEDs and supportive treatment	Recovered
Kour et al. (2023) [21]	22/F	9months pregnant	Dengue NS1 positive, IgM positive	Nil	Normotensive	AMS, blurring of vision with hemianopia	Nil	CT: Bilateral occipital and parietal cortex white matter hypodensities	AEDs and supportive treatment	Recovered
Kaur et al. (2022) [22]	8/F	Nil	Nil	Yes, hypotension	Hypotensive	Headache, seizure	Nil	MRI: T2W/FLAIR hyperintensities in subcortical regions of bilateral parieto-occipital lobes	AEDs and supportive treatment	Recovered
Cheo et al. (2021) [23]	15/M	Nil	Dengue NS1 positive; Dengue PCR: DENV2	Yes, compensated shock, respiratory distress	Normotensive	AMS	Nil	CT: Hypodensities at bilateral occipital regions and semiovale, predominantly white matter	Supportive treatment	Recovered
Chaudhuri et al. (2023) [24]	68/F	Nil	Dengue NS1 positive, IgM positive	Yes, hypotension	Hypotensive	Seizure, AMS, blurring of vision	Elevated protein, normal glucose and cell count	MRI: Symmetrical hyperintensities in the parieto-occipital subcortical white matter	Supportive treatment	Recovered
MAHMED. (2023) [25]	28/F	32weeks pregnant	Dengue NS1 positive	Nil	Normotensive	Seizure, AMS	White blood cell 3/ul, protein 0.2 g/L, glucose 0.4 g/L	MRI: Symmetrical parieto-occipital, fronto-parietal subcortical white matter hyperintensities	AEDs, supportive treatment	Recovered

### Pathophysiological considerations of dengue-associated PRES

The mechanisms linking DENV infection to PRES remain poorly understood. Classical PRES is thought to arise from failure of cerebral autoregulation during acute blood pressure fluctuations or from toxin/cytokine-mediated endothelial dysfunction leading to blood-brain barrier disruption and vasogenic edema [10, 11].

In dengue infection, systemic inflammation and endothelial injury with capillary leak are well known features, particularly in severe dengue infection during the critical phase. It is therefore plausible that cytokine release, endothelial dysfunction and increased vascular permeability may contribute PRES in certain individuals [7, 15]. Experimental studies have also demonstrated that dengue non-structural protein 1 (NS1) can interact with endothelial surfaces and disrupt the endothelial glycocalyx, potentially exacerbating vascular leakage [1, 20]. However, these observation are mainly derived from experimental laboratory models [7, 26], and the exact mechanisms underlying dengue-associated PRES remain uncertain.

### Strengths and limitations

The strengths of this case include comprehensive clinical assessment, detailed CSF analysis, serial neuroimaging, and longitudinal follow-up, allowing a thorough evaluation of overlapping dengue-related neurological processes.

Several limitations should be acknowledged. Due to the overlapping clinical and radiological features among dengue encephalitis, PRES and ischemic stroke, it would be difficult to determine the relative contribution of each process. Although the imaging pattern and radiological reversibility were more consistent with PRES, we can not entirely exclude the possibility of a rare atypical presentation of dengue encephalitis. As similar as most reported cases of dengue-associated PRES, the diagnosis relied on clinical and radiological evolution rather than histopathological confirmation.

### Conclusion

This case adds to the limited literature on dengue-associated PRES and emphasizes the need to consider overlapping neurological processes in patients with dengue-related CNS presentations, particularly in elderly patients in whom altered mental status is often prematurely attributed to delirium alone.

## Data Availability

The data supporting the findings of this study are available within the article.

## Abbreviations

DENV: Dengue virus
CNS: Central nervous system
PRES: Posterior reversible encephalopathy syndrome
AMS: Altered mental status
CSF: Cerebrospinal fluid
CT: Computed tomography
MRI: Magnetic resonance imaging
MCA: Middle cerebral artery
EEG: Electroencephalogram
MMSE: Mini-Mental State Examination
AEDs: Antiepileptic drugs
DIC: Disseminated Intravascular Coagulation
NS1: Non-structural protein 1
T2W: T2-weighted
FLAIR: Fluid-attenuated inversion recovery
